# Harmful Effects and Control Strategies of Aflatoxin B_1_ Produced by *Aspergillus flavus* and *Aspergillus parasiticus* Strains on Poultry: Review

**DOI:** 10.3390/toxins11030176

**Published:** 2019-03-23

**Authors:** Ahmed Mohamed Fouad, Dong Ruan, HebatAllah Kasem El-Senousey, Wei Chen, Shouqun Jiang, Chuntian Zheng

**Affiliations:** 1 Key Laboratory of Animal Nutrition and Feed Science (South China) of Ministry of Agriculture, State Key Laboratory of Livestock and Poultry Breeding, Guangdong Public Laboratory of Animal Breeding and Nutrition, Guangdong Key Laboratory of Animal Breeding and Nutrition, Institute of Animal Science, Guangdong Academy of Agricultural Sciences, Guangzhou 510640, China; ahmedmfouad@agr.cu.edu.eg (A.M.F.); donruan@126.com (D.R.); heba.agri_cairo@yahoo.com (H.K.E.-S.); cwei010230@163.com (W.C.); jsqun3100@hotmail.com (S.J.); 2 Department of Animal Production, Faculty of Agriculture, Cairo University, Giza 12613, Egypt

**Keywords:** aflatoxin B_1_, immunity, nutritional factor, productivity

## Abstract

The presence of aflatoxin B_1_ (AFB_1_) in poultry diets decreases the hatchability, hatchling weight, growth rate, meat and egg production, meat and egg quality, vaccination efficiency, as well as impairing the feed conversion ratio and increasing the susceptibility of birds to disease and mortality. AFB_1_ is transferred from poultry feed to eggs, meat, and other edible parts, representing a threat to the health of consumers because AFB_1_ is carcinogenic and implicated in human liver cancer. This review considers how AFB_1_ produced by *Aspergillus flavus* and *Aspergillus parasiticus* strains can affect the immune system, antioxidant defense system, digestive system, and reproductive system in poultry, as well as its effects on productivity and reproductive performance. Nutritional factors can offset the effects of AFB_1_ in poultry and, thus, it is necessary to identify and select suitable additives to address the problems caused by AFB_1_ in poultry.

## 1. Introduction

The allowance level for aflatoxins (AFs) is low in poultry feedstuffs compared with other mycotoxins and, thus, poultry feed is at a high risk of contamination with AFs. AFs are found in corn, which is one of the main sources of energy for poultry, as well as other feedstuffs, such as corn dried distiller’s grains with solubles, peanut meal, and cotton seed meal [1,2,3,4]. The growth of *Aspergillus flavus* (*A. flavus*) or *Aspergillus parasiticus* (*A. parasiticus*) in poultry feedstuffs is usually accompanied by the production of many toxic secondary metabolites, such as aflatoxins B_1_ (AFB_1_), B_2_ (AFB_2_), G_1_ (AFG_1_), and G_2_ (AFG_2_) [5]. Among these metabolites, AFB_1_ is the most dangerous and abundant mycotoxin [6,7]. The hazards from AFB_1_ in poultry are associated with low productivity and high susceptibility to diseases, which can have negative impacts on the poultry producer’s income as well as affecting human health [8,9,10,11]. AFB_1_ is recognized as being hepatotoxic, carcinogenic, and mutagenic [12]. AFB_1_ is the third most important cause of liver cancer, especially in sub-Saharan Africa and developing countries in Asia [13]. 

Eggs contain vitamins, minerals, and lipids, and are considered to be the cheapest source of animal protein [14,15], while poultry meat contains less fat and cholesterol than does ruminant meat [16,17,18]. Eggs, poultry meat, and/or products based on one or both, therefore, are consumed as part of the daily diet for many people [19,20,21,22]. Due to the harmful impacts of AFB_1_ on human health, the European Union has restricted the amount of AFB_1_ that can be present in food to 2.0 μg/kg [23]. AFB_1_ is transferred from poultry feed to eggs, meat, and other edible parts [24,25,26,27,28,29,30]. This review, therefore, considers the effects of AFB_1_ produced by *A. flavus* and *A. parasiticus* strains on poultry productivity through influencing the functionality of different organs, and examines how nutritional factors can help to address this problem.

## 2. Effects of Aflatoxin B_1_ Produced by *Aspergillus flavus* and *Aspergillus parasiticus* Strains on Poultry

### 2.1. Productivity

Table 1 summarizes the concentrations of AFB_1_ produced by *A. parasiticus* and *A. flavus* strains that detrimentally affected poultry productivity. The concentration of AFB_1_ that causes aflatoxicosis and impairs bird productivity depends on the fungal strain and the susceptibility of the bird species to AFB_1_. The susceptibility of bird species to AFB_1_ can be summarized as follows: aflotoxicosis occurred in breeders hens, broilers, quail, White Pekin ducks, and turkeys when they consumed diets containing 3000, 2000, 1500, 1100, and 500 μg AFB_1_/kg produced by *A. parasiticus* (NRRL 2999), respectively [31,32,33,34,35,36,37]. It has been reported [5] that the susceptibility of poultry species to AFB_1_ varies because each species has a different capacity for converting AFB_1_ to AFB_1_-8,9-epoxide (AFBO, the more toxic form) via the production of cytochrome P450 isozymes, which finally affect the formation and concentration of AFBO [38,39,40] and, thus, the concentrations of AFBO–DNA adducts (causing DNA lesions) [41,42]. It is unclear whether the same mechanism that explains the sensitivity of various poultry species to AFB_1_ can also explain the deleterious impacts of AFB_1_ produced by a specific fungal strain, even with a low concentrations, or if other mechanisms might be involved.

### 2.2. Egg and Meat Quality 

The biggest problem caused by AFB_1_ contamination in poultry production is not the economic loss due to poor eggshell quality and the reduced yolk color score, which can be readily observed and lead to rejection by consumers, but instead it is attributable to the AFB_1_ toxin, which is readily transferred from the diet to products (meat, eggs, and edible parts). Clearly, this toxin cannot be visually observed and specific tests are required for its detection to assess the possible threat to human health. It has been reported [34] that egg shell thickness and eggshell proportion were affected in quail that consumed a diet containing 1500 μg AFB_1_/kg produced by *A. parasiticus* (NRRL 2999). Similarly, reductions in the eggshell thickness and yolk color score, as well as high concentrations of AFB_1_ in the eggs and meat (1.4 and 18.0 μg/kg, respectively), were found when laying hens were fed a diet containing 2500 μg AFB_1_/kg produced by *A. parasiticus* (SRRC 148) from 2–40 weeks of age [9]. Moreover, the addition of 500 μg AFB_1_/kg produced by *A. parasiticus* (ATCC 15517) to the diet of hens from 15–67 weeks of age led to the accumulation of 3.5 and 18.2 μg AFB_1_/kg eggs at the beginning of the experiment and after one year of feeding, respectively [24]. However, increasing the concentration of added AFB_1_ obtained from *A. flavus* (NRRL 6540; CECT 2687) 10-fold compared with that used by Kim et al. [24] led to a lower accumulation of AFB_1_ (0.446 μg/kg) in the eggs after 21 days [51]. In addition, the AFB_1_ residue level in breast muscles from breeder hens aged 46 weeks was 0.03 μg/kg [51] compared with 18.0 μg/kg according to Pandey and Chauhan [9]. The different results obtained in these three previous studies [9,24,51] may be explained by the use of different fungal strains to produce AFB_1_, the bird ages, and/or the experimental duration. Indeed, feeding chicks for seven days with a diet containing 1600 μg AFB_1_/kg from *A. flavus* (NRRL 6540; CECT 2687) led to the deposition of 1.63, 0.49, and 0.41 μg/kg of AFB_1_ in muscles at 14, 21, and 28 days of age, respectively [10], thereby indicating that younger chicks accumulated more AFB_1_ than did older birds. 

Broilers fed a diet containing 40 μg AFB_1_/kg (produced by a local Chinese strain of *A. flavus*) for 21 days, 50 μg AFB_1_/kg (produced by *A. parasiticus*, NRRL 2999) for 28 days, 50 μg AFB_1_/kg (produced by a local Thailand strain of *A. flavus*) for 39 days, or 250 μg AFB_1_/kg (produced by *A. flavus*, KP 137700) for 39 days accumulated concentrations of 11.48, 0.40, 0.1, and 12.8 μg AFB_1_/kg in the liver, respectively [25,26,27,28]. The hepatic accumulation of AFB_1_ in broilers was 0.17 μg/kg after they consumed feed containing 1000 μg AFB_1_/kg in the pure crystal form for seven weeks [52], whereas a similar concentration of AFB_1_ produced by *A. flavus* (NRRL 3357) led to the deposition of 0.33 μg AFB_1_/kg in chicken livers after feeding the contaminated diet for 28 days [30]. In addition, increasing the level of AFB_1_ from a pure source five-fold (5000 μg AFB_1_/kg) for 18 days led to the accumulation of less AFB_1_ in the liver (0.11 μg/kg) [53] compared with the levels detected by Denli et al. [52] (0.17 μg AFB_1_/kg) and Rajput et al. (0.33 μg AFB_1_/kg) [30], thereby indicating that the concentration of AFB_1_ is not the only major factor that determines the effect of AFB_1_ and its accumulation in edible poultry parts; the fungal strain producing the toxin may, instead, be the main factor. In addition to the risks associated with the detection of AFB_1_ in the edible parts of poultry, the presence of malondialdehyde (MDA) may be more hazardous than AFB_1_ to human health. MDA is recognized as being a carcinogenic substrate because it reacts with DNA to induce mutations, which can lead to cancer, especially hepatic cancer [54,55]. In general, the concentrations of MDA in poultry meat and the edible parts may be elevated irrespective of whether the AFB_1_ level in the diet is low (22 μg/kg) or high (2500 μg/kg) [30,48,56,57]. 

### 2.3. Bones 

Tibial length, weight, strength, and mineralization are used to evaluate the bone quality in meat-type and egg-type birds [58,59]. In meat-type birds, bones are the main structures that will support the meat yield, so poor bone quality will lead to the appearance of leg problems during the finishing period, thereby hindering skeletal muscle development (constraining their genetic potential) and broken bones may occur after slaughter, adversely affecting carcass appearance and consumer acceptance [60]. In egg-type birds, almost one-third of the calcium (Ca) used for eggshell comes from bones because the Ca required for eggshell formation is not met solely by dietary sources [61]. Bones, therefore, represent calcium stores and poor bone quality is associated with bone weakness, low productivity, low eggshell quality, and economic losses [62]. Huff et al. [63] found that contamination of broiler diet during the first 3 weeks of age with 500 μg AFB_1_/kg generated by *A. parasiticus* (NRRL 2999) decreased tibial diameter and strength. Reduction of tibial strength in birds fed a diet containing AFB_1_ produced by *A. parasiticus* (NRRL 2999) may be from reduced deposition of Ca, phosphorus (P), zinc, and manganese in the tibia [64]. An association has been observed [65] between the concentration of AFB_1_ in the diet and the concentration of AFB_1_ in eggs. Therefore, AFB_1_ has been injected directly into fertilized eggs to save money, time, and effort when determining its effects. For instance, the injection of 0.04 μg AFB_1_/egg suppressed tibial growth (weight and length), which was linked to reduced hatchling weight and increased yolk sac weight [66]. Similar findings in terms of the tibial weight and length, embryo weight at 18 days of incubation, and the yolk sac weight were confirmed after injecting 0.05 μg AFB_1_ per egg [67]. The suppression of tibial growth could be due to the weak proliferation and hypertrophy of the growth plates, which are measured to evaluate bone development [66]. It was shown [68] that depressed skeletal development affected muscle development, where the embryo weight, and the weights of leg and breast muscle, decreased significantly at different stages of embryo development after the injection of 0.04 μg AFB_1_/egg; these were a consequence of suppressed cell proliferation and reduced number of myotube nuclei, thereby explaining the depressed muscle development and increased yolk sac weight in embryos from eggs injected with AFB_1_. The meat and egg yield will be affected in chicks that exhibit poor development during different stages of embryonic development. In broiler chickens that ingested a diet contaminated naturally with 82 and 134 μg AFB_1_/kg during the starter and grower phases [69], depressed growth rate and reduced tibial strength were associated with reduced ash, Ca, and P concentrations in the tibia. These were caused by suppressed production of 1,25-dihydroxycalciferol (which decreases Ca and P concentrations in blood and reduces tibial deposition of Ca and P) and the stimulation of parathyroid hormone (which activates osteoclasts to release calcium and organic components from bone and consequently weakening the bone). Reductions in weight gain, Ca concentrations, tibial weight, and tibial mineralization were found [70] in broilers fed a diet containing 2000 μg AFB_1_/kg produced by *A. parasiticus* (NRRL 2999) for 21 days.

### 2.4. Immune Organs 

Absolute and relative weights of immune organs are used to indirectly assess the immune status of birds; changes in their relative weights may result in altered in immune function. Table 2 summarizes the effects of AFB_1_ produced by *A. flavus* and *A. parasiticus* strains on the relative weights of the spleen, thymus, and bursa of Fabricius in poultry. In some studies [71], while the relative weights of the immune organs were not significantly affected by AFB_1_, histological changes in the organs were observed. Thus, the histological changes might not have been sufficient to cause significant reductions in the relative weights of the immune organs. In general, the suppression of lymphoid organ growth induced by AFB_1_ is related to reduced numbers of lymphocytes [72,73,74]. It has been reported [73,75] that the reduction in the relative weight of the bursa induced by AFB_1_ can be attributed to the decreased diameter of the lymphoid follicles and the reduced number of lymphocytes. In addition, increased relative weight of the spleen induced by AFB_1_ may be caused by the presence of congested red pulp in the organ [76], while reduced relative weight of the spleen caused by AFB_1_ may occur because its white pulp contains less lymphoid tissue [77]. These findings indicate that the lymphoid organs differ in terms of their sensitivity to AFB_1_, with the spleen being the most sensitive, followed by the bursa of Fabricius and thymus, possibly because the spleen receives and accumulates more AFB_1_ than the others [25]. AFB_1_ can suppress the activities of antioxidant enzymes and elevate content of MDA in the spleen, bursa, and thymus to cause oxidative damage, cell necrosis, and an increase in apoptosis [73,78,79]. This could account for AFB_1_ decreasing relative weights of the immune organs, thereby leading to their malfunction. It is not surprising, therefore, that significant declines in the production of antibodies, including IgA, IgG, and IgM, as well as the proportions of T and B lymphocytes, were found in broiler fed diets containing 40 μg AFB_1_/kg from *A. flavus* [25] and 1000 μg AFB_1_/kg produced by *A. flavus* (NRRL 3357) [30]. In addition, contaminating the maternal diet with 5000 μg AFB_1_/kg from *A. flavus* (NRRL 6540; CECT 2687) for three weeks significantly decreased the synthesis of IgA, IgG, and IgM in offspring chickens aged 21 days despite their being fed an AFB_1_-free diet [80]. Furthermore, the antibody titers against sheep red blood cells, Newcastle disease virus, and avian influenza (H5N1) were decreased by poultry diets contaminated with AFB_1_ [34,47,48,81,82].

### 2.5. Pancreas

The pancreas produces and secretes the digestive enzymes required to intestinally degrade feed and release nutrients to support the growth of birds, so they can express their genetic potential. A low concentration of AFB_1_ (20 μg/kg) produced by *A. flavus* (CICC 2219) in the diet of Cherry Valley ducks for six weeks led to a significant increase in the relative weight of the pancreas [50]. In contrast, 100 μg AFB_1_/kg from *A. parasiticus* (NRRL 2999) in the diet of broiler breeders for one month had no effect on the pancreas [84], but 300 μg AFB_1_/kg obtained from this same strain led to pancreatic hypertrophy [64]. The increased relative weight of the pancreas caused by AFB_1_ may be due to the high quantity of mature crystalline granules in the pancreatic cells [85]. The abnormal size of the pancreas in birds fed AFB_1_ may affect its functions, where the amylase, lipase, protease, chymotrypsin, and trypsin activities were elevated according to some studies [50,86,87,88], which would normally be expected to enhance the digestion of nutrients. Despite increased activities of digestive enzymes, the apparent digestibility of crude protein decreased without change in apparent digestibility of other nutrients in Cherry Valley ducks [50]; in White Pekin ducks, however, apparent ileal digestible energy decreased and the apparent ileal digestible nitrogen did not change when birds were fed a diet containing 200 μg AFB_1_/kg from *A. parasiticus* (NRRL 2999) for two weeks [89]. Indeed, the increased activities of digestive enzymes may be deceptive because oxidative damage and injury to the pancreas could occur, thereby compromising integrity of pancreatic cells leading to the release of proenzymes [87]. The percentage of nitrogen and dry matter stored in birds fed a diet containing AFB_1_ were unchanged but the birds lost weight [1,90,91], thereby confirming that the increased activities of digestive enzymes were related to a physiological problem. 

### 2.6. Intestine

The intestinal villus height, crypt depth, and the ratio of the villus height to the crypt depth (H/D) are measured to assess the ability of the intestine to absorb nutrients [92]. In laying hens, 1200 μg AFB_1_/kg produced by *A. parasiticus* (NRRL 2999) had no effect on the villus height but it reduced H/D ratio in the jejunum [90]. Villus height, crypt depth, and H/D were all reduced in laying quail fed a diet contaminated with 1500 μg AFB_1_/kg from *A. parasiticus* (NRRL 2999) [34]. In addition, dietary treatment with only 2.0 μg AFB_1_/kg synthesized by *A. parasiticus* (PTCC 5286) caused a significant reduction in the villus height, a significant increase in the crypt depth, and a decrease in the jejunal H/D in broilers [43,44]. In the jejunum, decreases in the number of absorptive cells, weakened cell integrity, lesions, increased apoptosis, and suppression of the cell cycle in phase G2/M were found when AFB_1_ was present in the diets of broilers [93,94,95,96,97,98]. These findings may be explained by the capacity of the intestine to accumulate AFB_1_; intestinal concentration of AFB_1_ 18 μg/kg when the dietary level was 22 μg/kg [29]. This may explain why AFB_1_ leads to abnormal development of the intestine and subsequent intestinal malfunctions. In the small intestine, lesions and reduced numbers of goblet cells that produce mucin 2 [99] could facilitate invasion of the intestine by harmful bacteria and adversely affect immunity. Thus, the populations of *Escherichia coli*, *Clostridium perfringens*, and Gram-negative bacteria increased in the ileal digesta of chickens fed a diet containing 40 μg AFB_1_/kg produced by *A. flavus* for 42 days [98]. However, feeding for 28 days with a diet containing 2.0 μg AFB_1_/kg produced by *A. parasiticus* (PTCC 5286) in broilers led to the population being dominated by *E. coli*, *Salmonella, Klebsiella*, and total Gram-negative bacteria [43,44]. In laying quail, treatment with feed containing 1500 μg AFB_1_/kg produced by *A. parasiticus* (NRRL 2999) for five weeks increased the numbers of coliforms, *Salmonella*, and *E. coli* in the cecum [34]. In addition, the numbers of IgA^+^ cells, and abundance of transcripts for antibodies (IgA, IgM, and IgG), as well as their production decreased in the small intestine of broilers fed a diet containing AFB_1_ [100,101]. The cecal tonsils are considered among the largest lymphoid organs in the gut-associated tissues of birds and they are linked with mucosal immunity. AFB_1_ led to the appearance of lesions in the absorptive cells and decreased numbers of lymphocytes in the lymphatic nodules of the cecal tonsils [102]. The numbers of IgA^+^ cells, T cells, and their subsets (CD3^+^, CD3^+^CD4^+^, and CD3^+^CD8^+^), as well as the transcripts for antibodies (IgA, IgM, and IgG) and cytokines (interleukin 2 (IL2), tumor necrosis factor alpha (TNFα), and interferon (IFN-γ)) were also reduced in the cecal tonsils of chickens after consuming feed contaminated with AFB_1_ [102,103]. The appearance of lesions, fewer absorptive cells, increases in harmful bacteria, suppression of mucosal immunity in the intestines of birds, and impaired intestinal functions could explain the retarded development of various organs in birds after consuming diets contaminated with AFB_1_. 

### 2.7. Liver

The liver is the main organ that processes mycotoxins, detoxifies them, and protects the body against their toxic effects. The liver is a central organ for lipid, protein, and amino acid metabolism, and their utilization [104,105], and is also involved in the hydroxylation of cholecalciferol to 25-hydroxycholecalciferol via 25-hydroxylase [106]. This intermediate is the precursor of 1,25-dihydroxy cholecalciferol, the most potent form of vitamin D_3_. The morphological and histological changes caused by AFB_1_ in the liver can be expected to result in functional changes. Table 3 summarizes the effects of AFB_1_ produced by *A. flavus* and *A. parasiticus* strains on the relative weight of the liver in poultry. Abnormal liver size may be associated with liver malfunctions. AFB_1_ can cause imbalanced lipid metabolism, promoting lipid deposition in the enlarged liver [107,108], repress the activity of antioxidant enzymes and anti-inflammatory cytokines, enhance lipid peroxidation and pro-inflammatory cytokines, and increase hepatocyte apoptosis [53,109,110,111,112,113]. The usual deleterious effects of AFB_1_ on hepatocytes result in high concentrations of aspartate aminotransferase and alanine aminotransferase in poultry blood after feeding diets containing AFB_1_ [37,50,71,81,114]. Aspartate aminotransferase (found in mitochondria) and alanine aminotransferase (found in the cytoplasm) are involved in hepatic protein metabolism, and they can determine the cell integrity [115,116]. Thus, the plasma content of total protein, albumin, globulin, triglycerides, and cholesterol decreased in poultry fed diets containing AFB_1_ [28,30,71,83,108,117,118], thereby indicating diminished protein and lipid biogenesis, which could account for reduced productivity of poultry fed such diets. 

### 2.8. Kidney

The kidney is involved in synthesizing the active form of vitamin D by converting 25-hydroxycholecalciferol into 1,25-hydroxycholecalciferol via 1-α-hydroxylase [106], as well as clearing blood of dangerous waste products of metabolism and participating in the maintenance of biochemical homeostasis in birds [118,119]. Due to these functions, the kidney is the main organ that accumulates AFB_1_ in poultry. In particular, the liver accumulated 8.3 μg AFB_1_/kg while the kidney accumulated 16.2 μg AFB_1_/kg when chicks were fed diets contaminated with 2500 μg AFB_1_/kg produced by *A. parasiticus* (NRRL 2999) from hatch to day 21 [120]. In broilers, the liver accumulated 11.5 μg AFB_1_/kg and the kidney accumulated 45.4 μg AFB_1_/kg when chicks consumed a diet containing 40 μg AFB_1_/kg produced by *A. flavus* during the first 21 days [25]. The experimental periods and bird ages were similar in these two studies [25,120], and it is interesting that the kidney accumulated more AFB_1_ (45.4 μg vs. 16.2 μg/kg) when the AFB_1_ concentration was lower in the diet (40 μg vs. 2500 μg/kg), although different fungal strains were used in the two studies. The capacity of the kidney to process and accumulate AFB_1_ is higher than that of the liver, thereby making it the main organ in birds for accumulating AFB_1_. As a consequence, it is one of the main organs exposed to oxidative damage from AFB_1_. The kidney contained higher levels of MDA than did liver (160 nmol vs. 70 nmol/g, and 133 nmol vs. 99 nmol/g) when the same strain of chicks with similar ages consumed diets containing 1000 and 150 μg AFB_1_/kg produced by *A. parasiticus* (NRRL 2999) for a similar experimental period [121,122]. The proportion of apoptotic cells was found to increase in the kidneys of broilers fed a diet containing AFB_1_ [123]. Although AFB_1_ increased the percentage of apoptotic renal cells, kidney enlargement also occurred [24,71,83,117,118,122,124], possibly due to increases in the number of mesangial cells and thickness of the glomerular basement membrane, and distension of the tubular epithelium cells as a consequence of granular degeneration [122,124,125]. It is not surprising, therefore, that the concentrations of creatinine and uric acid increased in the blood, reliable indicators of renal dysfunction [126,127], of birds that ingested feed contaminated with AFB_1_ [71,124]. Thus, various findings indicate that AFB_1_ causes kidney malfunction, which could in turn explain the reduced levels of 1, 25-dihydroxycalciferol, Ca, and P in the blood of birds fed diets containing AFB_1_ [24,69,83,118], thereby accounting for the poor bone mineralization, tibial bone quality, and eggshell quality noted earlier. Indeed, malfunctions of the intestine, liver, and kidney occurred concurrently in poultry that consumed diets contaminated with AFB_1_.

### 2.9. Reproductive Organs

As described above, reducing the daily feed intake is the first response observed in poultry that consume diets containing AFB_1_; this may be sufficient to reduce the relative weights of various organs, including those of the reproductive tract. Indeed, the abundance of vascular tissues surrounding the testes and their diameter declined, while the color of the testes changed from white to yellow, and the relative weight of the testes was lower when roosters were fed diets containing 5, 10, and 20 μg AFB_1_/kg produced by *A. parasiticus* (NRRL 2999) for eight weeks [128]. Similar results were obtained in male quail after consuming a diet containing 2500 μg AFB_1_/kg produced by *A. parasiticus* (PTCC 5286) for four weeks [48,129]. In addition, the concentration of testosterone in quail plasma that ingested only 2.5 μg AFB_1_/kg produced by *A. parasiticus* (NRRL 2999) for three weeks was reduced to almost one-third of that of the controls [130]. The same change was found [128] in roosters fed a diet containing 5 μg AFB_1_/kg from *A. parasiticus* (NRRL 2999) for eight weeks, spermatogenesis was suppressed with increased the production of abnormal spermatozoa. The performance of birds obtained by artificial insemination using semen produced by male birds that ingested AFB_1_ in their diet has not been tested but we consider that their fertility could be impaired. Reduced ovarian weights, suppressed follicle development, and the presence of only small follicles were reported in laying hens and quail after consuming diets containing 3300 μg AFB_1_/kg from natural contamination or 10 μg AFB_1_/kg produced by *A. parasiticus* (NRRL 2999) for three or four weeks, respectively [131,132]. Poults required longer (3–7 weeks or more) to reach sexual maturity when they ingested a diet containing AFB_1_ compared with those on an AFB_1_-free diet [9]. The deposition of AFB_1_ in the eggs of layer breeder hens started from the fifth day when they consumed a diet containing AFB_1_ [80]. In embryos, AFB_1_ can bind with DNA to induce mutations by altering some bases in the promoter sequences of growth hormone regulated gene1 [42]. This may explain the low hatchability, high percentage of defective embryos, and high proportion of embryonic mortality found in layer breeder hens when fed a diet containing AFB_1_ for three weeks [46]. Therefore, the reproductive systems of male and female birds are susceptible to the effects of AFB_1_. The low levels of AFB_1_ allowable in poultry diets in some countries, such as China (10 μg AFB_1_/kg diet) [1,81,86] and the European Union (20 μg AFB_1_/kg diet) [133], could adversely affect the development of the reproductive system in both sexes to subsequently suppress their fertility and reproduction. 

## 3. Nutritional Factors for Counteracting AFB_1_

### 3.1. Inorganic AFB_1_ Binders

Several materials have been tested as AFB_1_ binders. In chickens, adding 15 g of clinoptilolite, 5.0 g of hydrated sodium calcium aluminosilicate, or 5.0 to 7.5 g of bentonite/kg to a diet contaminated with AFB_1_ produced by *A. parasiticus* (NRRL 2999) at concentrations of 2000–2500 μg relieved the deleterious effects of AFB_1_ on performance [31,70,134,135], decreased the concentration of AFB_1_ from 8.3 μg to 1.5 μg/kg in liver [120], reduced extent of hepatic lesions, and increased protein synthesis [70]. Similarly, adding 7.5 g of bentonite/kg diet containing 600 μg AFB_1_/kg produced by *A. flavus* (NRRL 6540; CECT 2687) decreased the level of AFB_1_ from 1.21μg to 0.16 μg/kg in the liver [136]. In addition, adding 5.0 g of hydrated sodium calcium aluminosilicate/kg diet containing 2000 μg AFB_1_/kg from *A.parasiticus* (NRRL 2999) maintained the relative weight of the liver in broilers to that of birds on the AFB_1_-free diet [135]. In another study [25] with chickens fed a diet with 40 μg AFB_1_/kg produced by *A. flavus*, 3.0 g of hydrated sodium calcium aluminosilicate/kg decreased the hepatic accumulation of AFB_1_, increased the amount of AFB_1_ excreted, and reduced the relative weight of the liver, but it failed to maintain liver size to that with the AFB_1_-free diet. These findings suggest that the amount of the same AFB_1_ binder should be chosen according to the strain of fungus that produces AFB_1_ as well as the concentration of AFB_1_ in the diet. Thus, when the AFB_1_ produced by the same fungal strain and the animal model was not changed, the doses of different AFB_1_ binders that induced the same effect (binding AFB_1_ and protecting birds from its toxic effects) varied [31,60,135], probably due to differences in the efficiency of AFB_1_ binders. Therefore, a new option consisting of an AF nano-binder has been developed, where adding 2.5 g of nano-clay/kg diet contaminated with 110 μg AFB_1_/kg was more efficient at improving the productivity of turkeys by protecting the liver, kidney, and intestine, as well as for enhancing their functions compared with molecular clay [137,138]. In addition, adding nano-composite magnetic graphene oxide with chitosan (5.0 g/kg diet) decreased the concentration of AFB_1_ in the intestine from 18 μg to 6 μg/kg and increased body weight gain and FCR to the levels achieved with an AFB_1_-free diet when chickens consumed a diet contaminated with 22 μg AFB_1_/kg produced by *A. parasiticus* (FRR 2999) [29]. Nanoproducts are available on a commercial scale but their impacts on human health and the environment are not well understood; thus, alternative solutions are needed. 

### 3.2. Organic AFB_1_ Binders

#### 3.2.1. Yeast

It has been reported that the β-1-3 glucane and mannoproteins found in yeast cell walls can bind AFB_1_ [139]. In breeder hens, adding 100 mg of yeast cell walls (containing 26 g of β-glucan and 15 g of mannan-oligosaccharides/100 g) normalized the secretion of digestive enzymes such as lipase and chymotrypsin after birds consumed feed containing 1000 μg AFB_1_/kg formed by *A. parasiticus* (NRRL 2999) [84]. Yeast cell walls containing D-glucose (48.3%) and D-mannose (32.3%) at a level of 0.5 g/kg failed to affect the performance or immunity in chickens that consumed a diet containing 40 μg AFB_1_/kg produced by *A. flavus* [45]. Adding 1.5 g/kg *Saccharomyces cerevisiae* yeast cell walls to a diet containing 350 μg AFB_1_/kg in broilers restored the daily weight gain as well as enhancing the FCR and antibody production relative to birds given an AFB_1_-free diet [72]. Moreover, in broilers, the addition of 1.0 g/kg yeast (*Pichia kudriavzevii*) to a diet that contained 100 μg AFB_1_/kg produced by *A. parasiticus* (NRRL 2999) increased the final body weight and carcass yield, and reduced the concentration of AFB_1_ in the liver by 14% [140]. However, adding 0.5 or 1.0 g/kg of yeast (*Trichosporon mycotoxinivorans*) to diets contaminated with 100 or 600 μg AFB_1_/kg produced by *A. flavus* (NRRL 6540; CECT 2687) failed to bind the AFs [136]. Yeast was also effective when added to drinking water with 5 × 10^9^ of *Saccharomyces cerevisiae* CECT 1891 cells/L drinking water for broilers fed a diet containing 1200 μg AFB_1_/kg produced by *A. parasiticus* (NRRL 2999), where it reduced the increase in the size of the liver, improved protein synthesis, and restored the growth performance [141]. In addition, brewing waste containing yeast cell walls counteracted AFB_1_ when 10 g/kg cell walls was added to a broiler diet contaminated with 2000 μg AFB_1_/kg produced by *A. parasiticus* (NRRL 2999); improved protein synthesis and the Ca level in the blood, decreased hepatic lesions, and increased growth rate were observed [142]. These results indicate that different yeasts with different cell wall components account for the varying results obtained in previous studies with different fungal strain and resistance of birds.

#### 3.2.2. Probiotic

Other microorganisms, particularly bacteria, can also have important effects, for example 1.0 g/kg of probiotic (commercial product) added to a diet containing 250 μg AFB_1_/kg produced by *A. flavus* (KP137700) improved the antioxidant status, liver function, protein synthesis, and productive performance of broiler chickens compared with an AFB_1_-free diet. This treatment reduced the level of AFB_1_ from 12.8 to 2.9 μg/kg in the liver compared with the AFB_1_-contaminated diet [28]. Moreover, mixing similar amounts of *Lactobacillus acidophilus*, *Lactobacillus plantarum*, and *Enterococcus faecium*, and adding them to the diet at a level of 1.5 × 10^10^ cfu/kg enhanced the digestibility of nutrients and antibody production, decreased the concentrations of AFB_1_ in the liver (from 11.5–2.2 μg/kg) and immune organs, maintained the normal relative weights of the liver and immune organs, and improved the performance of broilers fed a diet contaminated with 40 μg AFB_1_/kg produced by *A. flavus* [25]. Similarly, *Bacillus subtilis* (ANSB060) could detoxify AFB_1_, where 2.0 g/kg added to a broiler diet containing 70 μg AFB_1_/kg produced from moldy peanut meal decreased the accumulation of AFB_1_ from 7 to 1.5 μg/kg in the intestine and from 0.24 to 0.09 μg/kg in the liver, as well as reducing hepatic lipid peroxidation and enhancing liver functions, average daily gain, and FCR [143,144]. In ducks, adding 1.0 g/kg of *Bacillus subtilis* (ANSB060) to the diet was sufficient to counteract the toxicity of 22 μg AFB_1_/kg formed from moldy corn, where it increased the activity of antioxidant enzymes, improved FCR, and reduced AFB_1_ concentration from 0.12 to 0.06 μg/kg in the liver [56]. In laying hens, a combination of two strains of *Bacillus subtilis* (ANSB060 and ANSB01G) reduced the amount of the bacterium required to neutralize AFB_1_; 1.0 g/kg of the mixture improved laying performance, and delayed the appearance and concentration of AFB_1_ in the eggs, when hens consumed a diet contaminated with 123 μg AFB_1_/kg formed from moldy peanuts and corn meal [145]. In quail, 10^8^ cfu of *Berevibacillus laterosporus*/mL of drinking water decreased hepatic necrosis and enhanced liver function, protein production, antibody levels, growth rate, and meat yield when fed a diet containing 2500 μg AFB_1_/kg produced by *A. parasiticus* (PTCC 5286) [47]. In broiler chickens, adding 10^8^ cfu/mL of *Lactobacillus plantarum 299v* to drinking water increased the activity of antioxidant enzymes, reduced lipid peroxidation, and enhanced protein synthesis and the final body weight gain when they fed a diet containing 200–2000 μg AFB_1_/kg synthesized by *A. parasiticus* (PTCC 5286) [146]. Quail are more sensitive to AFB_1_ than are broiler chickens [5,38], but when the fungal strain that produced AFB_1_ and concentrations of the two probiotic bacteria were the same, it was shown [47] that *Berevibacillus laterosporus* counteracted the toxicity of 2500 μg AFB_1_, whereas *Lactobacillus plantarum 299v* [146] could only efficiently counteract the toxicity of 200 μg AFB_1_. These and other findings, therefore, suggest that the presence of one or more probiotic bacterial strains at particular optimized concentrations can efficiently counteract the toxicity of AFB_1_, again varying with the fungal strain and susceptibility of specific birds. Probiotic bacteria (organic AFB_1_ binder) and clay (inorganic AFB_1_ binder) [25] but not yeast cell walls (organic AFB_1_ binder) [45] restored the productivity and immunity in broilers fed diets containing the same level of AFB_1_ produced from the same fungus during the same experimental period, but the probiotic bacteria (organic AFB_1_ binder) were more efficient than clay (inorganic AFB_1_ binder) in reducing the concentrations of AFB_1_ in the liver, kidney, and lymphoid organs. 

### 3.3. Antioxidants

**Figure 1:**
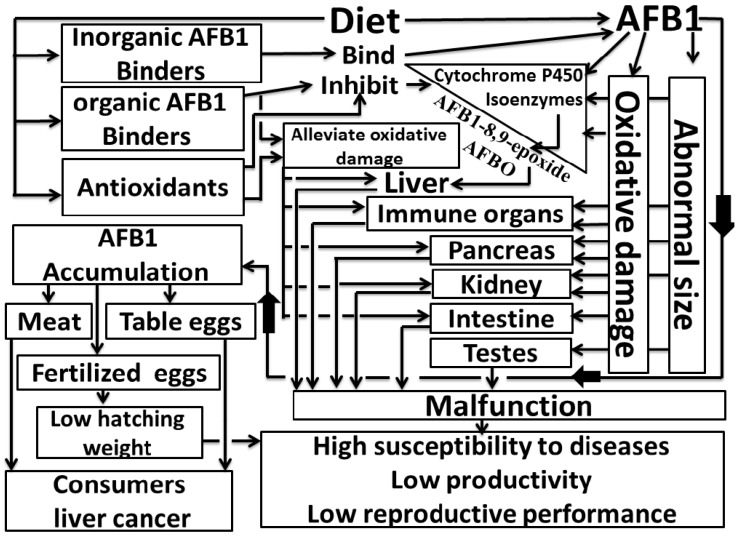
Impacts of aflatoxin B_1_ (AFB_1_) on poultry organs functions, productivity, and consumer health, and nutritional factors that might mitigate these impacts. The presence of AFB_1_ in the diet of poultry leads to body organs with abnormal sizes, stimulates the production of cytochrome P450 isoenzymes to convert AFB_1_ into AFB_1_-8,9-expoxide (AFBO; as the more toxic form of AFB_1_), oxidative damage, and organ malfunctions, which led to low productivity, decreased reproductive performance, high susceptibility to diseases, and the accumulation of AFB_1_ in eggs and meat, which can be harmful to the health of consumers. Adding inorganic AFB_1_ binders can bind AFB_1_ and reducing the accumulation of AFB_1_ in eggs and meat according to their efficiency. Organic AFB_1_ binders, such as probiotics, can bind or absorb AFB_1_ to decrease the conversion of AFB_1_ into AFBO by suppressing cytochrome P450 isoenzymes, as well as alleviating oxidative damage to organs and reducing the accumulation of AFB_1_ in eggs and meat. The addition of antioxidants, such as selenium and curcumin, can decrease the conversion of AFB_1_ into AFBO by suppressing cytochrome P450 isoenzymes and alleviate oxidative damage to organs.

As discussed above, birds exposed to AFB_1_ toxicity reduce their feed consumption, and thereby may not consume adequate amounts of dietary antioxidants for the effective functioning of the antioxidant defense system. In addition, AFB_1_ activates the formation of reactive oxygen species and free radicals to higher levels than the body can eliminate, thereby increasing lipid peroxidation and causing oxidative damage to most of the bodily organs. Therefore, providing antioxidants to poultry exposed to AFB_1_ might help support the antioxidant defense system, and improve productivity of poultry. In particular, adding 0.4 mg/kg selenium (Se) to diets enhanced the antioxidant defense system in the lymphoid organs [79,147,148,149,150], jejunum [97], and kidney [125], and protected against oxidative damage when chickens were fed diets containing 600 μg of AFB_1_/kg. Adding 300 μg/kg of alpha-lipoic acid alleviated the oxidative damage induced in the liver and kidney by AFB_1_ 74 μg/kg produced by moldy peanut meal and 300 μg/kg produced by *A. parasiticus* (NRRL 2999) [77,151], where it restored the levels of IL-6, IFN-γ, and TNFα in the blood, and their transcript abundance in the liver of chickens [112]. The addition of 300 mL of *Urtica diocia* seed extract/kg of diet containing 1000 μg AFB_1_/kg produced by *A. parasiticus* (NRRL 2999) enhanced the antioxidant status, reduced hepatic and renal lipid peroxidation, and decreased the reduction in final body weight due to AFB_1_ [122]. Including 250 mg of grape seed proanthocyanidin extract/kg in a diet containing 1000 μg AFB_1_/kg produced by *A. flavus* (NRRL 3357) increased the antioxidant enzyme activity, decreased lipid peroxidation, reduced the accumulation of AFB_1_ (0.35 vs. 0.18 μg/kg) in the liver, improved the synthesis of proteins including IgA, IgG, and IgM, and mitigated the reduced productivity of broiler chickens [30]. In addition, adding 74 mg/kg curcuminoids or 150 mg/kg curcumin to a broiler diet containing 1000 μg AFB_1_/kg produced by *A. parasiticus* (NRRL 2999) or 100 μg AFB_1_/kg as the pure crystal form reduced the increase in the relative weight of the liver and decreased the levels of alanine aminotransferase, aspartate aminotransferase, and lipid peroxidation, increased the antioxidant capacity and protein production, and offset the reduced average daily gain [109,152]. These results show that antioxidants are required by poultry exposed to AFB_1_ in order to enhance the efficiency of the antioxidant defense system; this might involve altering AFB_1_ metabolism to alleviate its toxicity. Thus, adding an antioxidant such as grape seed proanthocyanidin extract decreased the accumulation of AFB_1_ in the liver from 0.35 to 0.18 μg/kg [30]. Curcumin or selenium could suppress the transcription and activities of cytochrome P450 isozymes (essential enzymes for converting AFB_1_ into the more toxic form (AFBO)), where they decreased the levels of 8-hydroxydeoxyguanosine (which can destroy DNA) and the formation of AFBO–DNA adducts in the livers of chickens exposed to AFB_1_ in the diet [152,153]. Probiotics can improve the antioxidant status in broilers exposed to AFB_1_ by binding the toxin to decrease the hepatic formation of its more toxic form (AFBO) by downregulating transcription of cytochrome P450 isozymes [28]. Therefore, adding antioxidants and an AFB_1_ binder together could be more effective than adding individual treatments for overcoming the effects of AFB_1_ in poultry. However, dietary supplementation with 7.5 g/kg bentonite clay alone in a diet containing 2000 μg AFB_1_/kg produced by *A. parasiticus* (NRRL 2999) was better than a combination of 200 mg curcuminoids and 7.5 g/kg bentonite clay [70], although the combination of 74 mg curcuminoids and 5 g/kg hydrated sodium calcium aluminosilicate in a diet containing 1000 μg AFB_1_/kg produced by *A. parasiticus* (NRRL 2999) was better than adding 5 g/kg hydrated sodium calcium aluminosilicate in broilers [154]. The concentrations of curcuminoids and AFB_1_ binder, as well as the concentration of AFB_1_ in the diets differed in these studies, which could explain the different results obtained. The concentration of AFB_1_, the fungal strain that produces AFB_1_, and the efficiency of the AFB_1_ binder with or without added antioxidants all should be considered in further studies to determine the best methods for eliminating AFB_1_. However, Table 4 summarizes the concentrations of some additives used in poultry diets to counteract the toxicity of AFB_1_. Figure 1 summarizes the impacts of AFB_1_ on the functions of organs and productivity in poultry, as well as on the health of consumers, and the nutritional factors that might mitigate these impacts. 

## 4. Conclusions 

The concentration of AFB_1_ is a key factor related to the occurrence of aflatoxicosis in poultry but the fungal strain that produces AFB_1_ should also be considered. In particular, AFB_1_ produced by specific fungal strains can have highly deleterious impacts on poultry productivity even when concentration of the toxin is low. The reductions in productivity and reproductive performance induced in poultry by AFB_1_ are the consequence of malfunctions in most of the organs in poultry due to AFB_1_. Nutritional factors such as inorganic and organic AFB_1_ binders, as well as antioxidants, vary in terms of their efficiency and the mechanism involved when counteracting the deleterious impacts of AFB_1_ on poultry. In particular, binding AFB_1_, decreasing the formation of AFBO, maintaining a strong antioxidant defense system, protecting the organs against oxidative damage, and maintaining organ functions should be considered when selecting anti-AFB_1_ additives. It is necessary to develop new additives or combinations to more efficiently counteract the deleterious effects of AFB_1_ and restore poultry productivity.

## Figures and Tables

**Table 1:** Reported levels of aflatoxin B_1_ that impair poultry productivity.

Bird	Aflatoxin Dose *	Fungal Strain	Reference
Chickens	2000	*A. parasiticus* (NRLL 2999)	[31]
Chickens	22	*A. parasiticus* (FRR 2999)	[29]
Chickens	2	*A. parasiticus* (PTCC 5286)	[43,44]
Chickens	1000	*A. flavus* (NRRL 3357)	[30]
Chickens	250	*A. flavus* (KP137700)	[28]
Chickens	40	*A. flavus* (Chinese isolate)	[45]
Laying hens	2500	*A. parasiticus* (SRRC 148)	[9]
Laying hens	500	*A. parasiticus* (ATCC 15517)	[24]
Breeder hens	3000	*A. parasiticus* (NRRL 2999)	[32]
Breeder hens	500	*A. flavus* (NRRL 6540; CECT 2687)	[46]
Quail	2500	*A. parasiticus* (PTCC 5286)	[47,48]
Quail	1500	*A. parasiticus* (NRRL 2999)	[33,34]
Quail	500	*A. flavus*	[49]
Ducks	1100	*A. parasiticus* (NRRL 2999)	[35]
Ducks	20	*A. flavus* (CICC 2219)	[50]
Turkeys	500	*A. parasiticus* (NRRL 2999)	[36,37]

* Aflatoxin dose (μg /kg).

**Table 2:** Harmful effects of aflatoxin B_1_ on immune organs in poultry.

Bird	Aflatoxin Dose (μg/kg)	Fungal Strain	Relative Weights of Organs			Reference
			Spleen	Bursa	Thymus	
Chickens	40	*A. flavus* (Chinese isolate)	+	−	−	[25]
Chickens	22	*A. parasiticus* (FRR 2999)	+	±	ND	[29]
Chickens	4000	*A. parasiticus* (NRRL 2999)	+	ND	ND	[83]
Offspring of breeder hens	5000	*A. flavus* (NRRL 6540 CECT 2687)	−	−	±	[80]
Turkeys	330	*A. flavus* (UNIGRAS 1231)	±	±	ND	[71]
Turkeys	500	*A. parasiticus* (NRRL 2999)	±	±	ND	[37]

Abbreviations: + increase; − decrease; ± no effect; ND, not determined.

**Table 3:** Harmful effects of aflatoxin B_1_ on liver in poultry.

Bird	Aflatoxin Dose (μg/kg)	Fungal Strain	Relative Weight of Liver	Reference
Chickens	40	*A. flavus* (Chinese isolate)	+	[25]
Chickens	250	*A. flavus* (KP137700)	+	[28]
Chickens	1000	*A. flavus* (NRRL 3357)	+	[30]
Ducks	20	*A. flavus* (CICC 2219)	+	[50]
Ducks	1100	*A. parasiticus* (NRRL 2999)	±	[35]
Turkeys	330	*A. flavus* (UNIGRAS 1231)	−	[71]
Turkeys	500	*A. parasiticus* (NRRL 2999)	+	[37]
Laying hens	500	A. *parasiticus* (ATCC 15517)	+	[24]
Laying hens	1000	*A. parasiticus*	+	[107]
Laying hens	2500	*A. parasiticus* (SRRC 148)	+	[9]
Quail	2500	*A. parasiticus* (PTCC 5286)	+	[48]

Abbreviations: + increase; − decrease; ± no effect.

**Table 4:** Some additives used in poultry diets to counteract the toxicity of aflatoxin B_1_.

Item	Amount (g/kg)	Aflatoxin Dose (μg/kg)	Bird	Reference
Clinoptilolite	15.0	2500 (*A. parasiticus* NRRL 2999)	chickens	[134]
Hydrated sodium calcium aluminosilicate	5.0	2000 (*A. parasiticus* NRRL 2999)	chickens	[135]
Hydrated sodium calcium aluminosilicate	3.0	40 (*A. flavus*)	chickens	[25]
Bentonite	7.5	2000 (*A. parasiticus* NRRL 2999)	chickens	[70]
Bentonite	7.5	600 (*A. flavus* NRRL 6540; CECT 2687)	chickens	[136]
Nano-composite magnetic graphene oxide with chitosan	5.0	22 (*A. parasiticus* FRR 2999)	chickens	[29]
Yeast cell walls ^1^	1.5	350 (naturally contaminated)	chickens	[72]
Yeast ^2^	1.0	100 (*A. parasiticus* NRRL 2999)	chickens	[140]
Probiotic ^3^	1.0	250 (*A.flavus* KP137700)	chickens	[28]
Probiotic ^4^	2.0	70 (naturally contaminated)	chickens	[143,144]
Probiotic ^5^	1.0	22 (naturally contaminated)	ducks	[56]
Probiotic ^6^	1.0	123 (naturally contaminated)	hens	[145]
Alpha-lipoic acid	300 ^a^	300 (*A. parasiticus* NRRL 2999)	chickens	[77]
Urtica diocia seed extract	300 ^b^	1000 (*A. parasiticus* NRRL 2999)	chickens	[122]
Grape seed proanthocyanidin extract	250 ^a^	1000 *(A. flavus* NRRL 3357)	chickens	[30]
Curcuminoids	74.0 ^a^	1000 (*A. parasiticus* NRRL 2999)	chickens	[154]

Yeast wall cells ^1^ (*Saccharomyces cerevisiae*); Yeast ^2^ (*Pichia kudriavzevii*); Probiotic ^3^ (commercial product); Probiotic ^4^ (*Bacillus subtilis* ANSB060); Probiotic ^5^ (*Bacillus subtilis* ANSB060); Probioti c^6^ (*Bacillus subtilis* ANSB060 and *Bacillus subtilis* ANSB01G). ^a^ (mg/kg); ^b^ (mL/kg).
